# The Effect of Deoxynivalenol on Selected Populations of Immunocompetent Cells in Porcine Blood—A Preliminary Study

**DOI:** 10.3390/molecules22050691

**Published:** 2017-04-26

**Authors:** Michał Dąbrowski, Ewa Jakimiuk, Mirosław Baranowski, Magdalena Gajęcka, Łukasz Zielonka, Maciej Tadeusz Gajęcki

**Affiliations:** Department of Veterinary Prevention and Feed Hygiene, Faculty of Veterinary Medicine, University of Warmia and Mazury in Olsztyn, 10-719 Olsztyn, Poland; michal.dabrowski@uwm.edu.pl (M.D.); ewa.jakimiuk@uwm.edu.pl (E.J.); kerim@uwm.edu.pl (M.B.); mgaja@uwm.edu.pl (M.G.); gajecki@uwm.edu.pl (M.T.G.)

**Keywords:** deoxynivalenol, flow cytometry, lymphocytes, dendritic cells, swine

## Abstract

Deoxynivalenol (DON) is one of the most prevalent mycotoxins in Europe. Pigs are an animal species that is most susceptible to this mycotoxin. Deoxynivalenol causes significant losses in pig production by lowering feed intake, decreasing daily weight gains, disrupting immune responses, and increasing susceptibility to diseases. The aim of this experiment was to determine the influence of feed contaminated with DON at concentrations insignificantly higher than recommended by the European Commission (900 µg/kg). The experimental feed contained 1008 μg DON/kg. The experiment was performed on eight weaners from the same litter. The animals were randomly divided into two groups: an experimental group (M, *n* = 4) fed contaminated feed and a control group (C, *n* = 4) administered feed free of mycotoxins. The experiment lasted for six weeks, and peripheral blood samples were collected from the animals for analyses of selected morphological parameters and changes in the percentages of CD4^+^8^−^, CD4^−^8^+^, and CD4^+^8^+^ lymphocytes and antigen-presenting cells (APC) with CD14^+^172^+^ (monocytes), CD172a^high^4^−^14^−^ (conventional dendritic cells, cDC), and CD172a^dim^4^+^14^−^ (plasmacytoid dendritic cells, pDC) phenotypes. The morphological parameters of porcine blood samples were determined by flow cytometry with non-fluorescent particle-size calibration standards, and no differences were observed between groups M and C. An immunophenotyping analysis of lymphocytes and dendritic cells (DC) revealed an increase in the percentage of CD4^+^8^−^, CD172a^high^4^−^14^−^, and CD172a^dim^4^+^14^−^ cells, and a decrease in the number of CD4^−^8^+^ cells in group M. The results of this experiment suggest that prolonged exposure to low doses of DON can change the proportions of immunocompetent cells (a shift towards humoral immunity), without affecting their overall counts.

## 1. Introduction

Deoxynivalenol (DON) is a secondary metabolite of fungi of the genus *Fusarium*. It belongs to group B trichothecenes which are produced in cereals at low temperatures and high humidity in the field or during storage. Trichothecenes are strongly toxic for humans and animals. Pigs are an animal species that is most susceptible to DON, and even very low doses of the mycotoxin provoke histopathological changes in the porcine digestive tract and liver [1]. High doses of DON can induce nausea, vomiting, gastric disorders, dizziness, headaches, and diarrhea [2]. Deoxynivalenol causes significant losses in pig production by lowering feed intake and body weight gains [3]. Recent studies have focused on DON’s influence on the immune system and immune responses in animals exposed to this mycotoxin. Deoxynivalenol induces seemingly contradictory effects. Low doses of DON modulate the immune system, whereas high doses lead to immunosuppression [4]. It should be noted that DON isolated from naturally-contaminated grain which had been colonized by *Fusarium* fungi exerts stronger toxic effects than chemically-synthesized DON [5]. The immunosuppressive effects of DON include reduced activity of T and B cells, decreased production of antibodies, and impaired function of macrophages and neutrophils [6]. On the other hand, DON binds with eukaryotic ribosomes and induces a ribotoxic stress response which is responsible for the phosphorylation of mitogen-activated protein kinases (MAPK) ERK ½, JNK, and p38 [7]. Activated MAPKs stimulate the expression of mRNA and the secretion of proinflammatory cytokines IL-2, IL-4, and IL-5 [8]. Previous research has shown that DON may increase total serum IgA levels and the concentrations of ovalbumin (OVA)-specific IgA and IgG in animals immunized with OVA [9,10].

Deoxynivalenol is one of the most prevalent mycotoxins in cereal products and cereal-based feeds manufactured in the temperate region of Europe. Monbaliu et al. [11] analyzed 82 samples of feed for sows, wheat and maize, and detected DON in 63% of the samples in a concentration range of 74 to 9528 µg/kg. Rodrigues and Naehrer [12] evaluated a representative number of 2036 feed samples, and DON was identified in 58% of cases at a concentration of up to 49,000 µg/kg. According to a scientific report of the European Food Safety Authority (EFSA), the recommended safe level for DON (900 µg/kg) was exceeded in 6.9% of 259 analyzed samples of complete diets for pigs [13].

In the European Union, the guidance values for DON in complete and complementary feeding stuffs for pigs are set by Commission Recommendation No. 2006/576/EC [14]. Based on the above guidelines, DON levels may not exceed 900 µg/kg of feed. The maximum allowable dose of DON was set based on the opinion of the Scientific Panel on Contaminants in the Food Chain of the EFSA [15]. The above document was drafted based on numerous research studies, most of which (107 of 155) were conducted in the 1980s and the 1990s. It should also be noted that many of these studies were performed on farm-raised animals, which are characterized by high phenotypic and genotypic variation that may contribute to considerable differences in sensitivity to DON, high standard deviation, and errors in the interpretation of DON’s influence on pigs. In recent years, advanced detection techniques have been deployed to detect subtle changes induced by low mycotoxin doses. Contemporary methods support the identification of cell populations or even cell structures that are highly susceptible to mycotoxins.

The aim of this study was to determine the influence of feed naturally contaminated with DON at a concentration similar to that recommended by the European Commission [14] on morphological and selected immunological parameters (antigen-presenting cells (APC), changes in lymphocyte subpopulations) in the peripheral blood of full-sibling pigs.

## 2. Results

Feed naturally contaminated with mycotoxins was supplied by a commercial feed producer. The presence of DON, ZEN, fumonisins B_1_ and B_2_ (FB_1_ and FB_2_), and ochratoxin A (OTA) was determined by HPLC-UV (high-performance liquid chromatography coupled to UV–vis detection). Feed was contaminated with 1008 μg/kg DON. The remaining mycotoxins were below the limit of detection (LOD).

The feed administered to control group pigs was supplied by the same producer. It was also analyzed for the presence of DON, ZEN, FB_1_, FB_2_, and OTA, and the results were below LOD values. The proximate chemical composition of diets fed to pigs in groups C and M is presented in Table 1.

**Figure 1:**
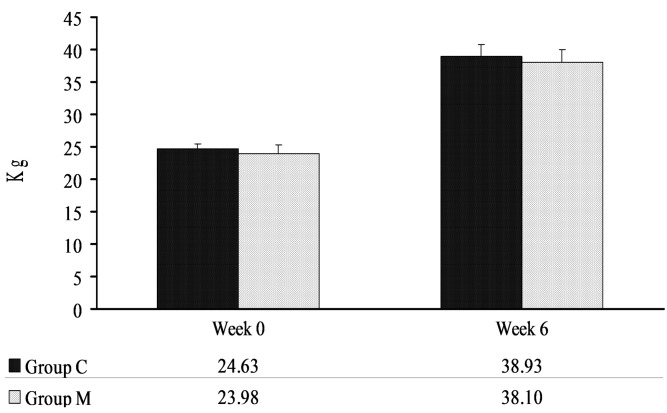
Body weights of pigs in weeks 0 and 6. The results were expressed as $\bar{x}$ and SD.

No differences were found between groups in the body weights of pigs determined in weeks 0 and 6 (Figure 1). The average daily weight gains over a period of six weeks reached 0.340 kg/pig and 0.336 kg/pig in groups C and M, respectively.

The morphological parameters of porcine blood samples were determined by flow cytometry with non-fluorescent particle-size calibration standards, and no differences were observed between groups M and C. The results of morphological analysis were expressed as means and SD, including white blood cell counts, platelet counts, and mean platelet volume (Table 2), and an erythrogram (Table 3).

**Figure 2:**
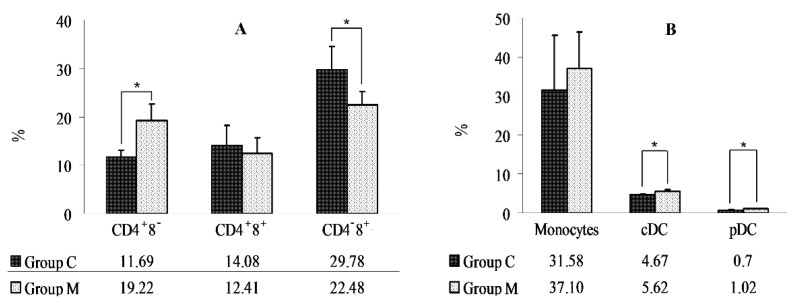
(**A**) Changes in the percentage of CD4^+^8^−^, CD4^+^8^+^, and CD4^−^8^+^ cells in groups M and C; (**B**) Changes in the percentage of monocytes, cDCs, and pDCs in groups M and C. The results were expressed as $\bar{x}$ and SD. *: statistically significant differences at *p* ≤ 0.05.

Lymphocyte subpopulations were analyzed for the presence of CD4 and CD8 surface glycoproteins, and an increase in the percentage of CD4^+^8^−^ cells and a decrease in the percentage of CD4-8+ cells were noted in group M (Figure 2A).

A cytometric analysis of the percentage of monocytes (CD14^+^), cDCs (CD172a^high^4^−^14^−^), and pDCs (CD172a^dim^4^+^14^−^) revealed an increase in cDC and pDC populations in group M (Figure 2B).

## 3. Discussion

The objective of the present experiment was to determine the effect of prolonged exposure to low doses of deoxynivalenol on blood cell morphology and the size of different subpopulations of immunocompetent cells.

A morphological analysis of blood cells did not reveal any differences between groups. The morphological parameters of red blood cells, white blood cells, and thrombocytes were within the reference ranges [16]. An immunophenotyping analysis of lymphocyte subpopulations demonstrated an increase in the relative number of CD4^+^8^−^ cells and a decrease in the number of CD4^−^8^+^ cells in pigs exposed to DON. The percentage of cDCs and pDCs increased in group M.

Blood morphology analyses are performed routinely in clinical diagnostic practice. Analyses of the size and proportions of different blood cell populations support rapid and non-invasive evaluations of the patients’ health. In the literature, mycotoxins have been found to exert varied effects on blood cell morphology, depending on the type of mycotoxin or its dose. Evaluations of DON’s influence on blood morphology did not support the determination of changes characteristic of this mycotoxin. Zielonka et al. [17] demonstrated that low doses of DON (12 µg/kg BW) can induce significant changes in red blood cell (RBC) counts, mean corpuscular volume (MCV), mean corpuscular hemoglobin concentration (MCHC), platelet (PLT) counts, mean platelet volume (MPV), and white blood cell (WBC) counts. Those changes were induced by synthetic DON, and fluctuations in the measured values were observed during multiple blood morphology analyses. In a study by Modra et al. [18], exposure to a DON dose of 2 mg/kg of naturally-contaminated feed lowered MCV values. The changes in MCV were observed in the last (fourth) week of the experiment, i.e., after prolonged exposure. Perlusky et al. [3] demonstrated that a DON dose of 3 mg/kg of feed can increase RBC, PLT, and hematocrit (HCT) values. Those changes were accompanied by decreased feed intake, which suggests that they could result from malnutrition. In a study by Accensi et al. [19], a DON dose of 840 μg/kg of feed did not induce any changes in the morphological or biochemical parameters of pigs exposed to the mycotoxin, but an increase in serum IgA levels was noted. Swamy et al. [20] did not report any changes in the blood morphology of pigs whose diets were highly contaminated with DON (5.8 mg/kg of feed), but the percentage of CD4^+^8^−^ cells increased relative to the pair-fed group. This could suggest that the activity of the immune system was affected by DON rather than the restricted feed ration. Rotter et al. [21] also observed that diets with increasing concentrations of DON (750, 1500, and 3000 µg/kg of feed) raised neutrophil counts and, consequently, total leukocyte counts in young swine. Neutrophils were also highly sensitive to DON in an in vitro study. Neutrophils suspended in a medium containing 0.5–10 µM DON exhibited lower chemotaxis, lower phagocytic activity, and lower ability to secrete IL-8 [22]. The reference values of morphological parameters in porcine blood [16] indicate that different cell populations in peripheral blood span a wide physiological range. The above points to considerable variations in the reactivity of the porcine immune system, which suggests that subtle changes induced by low mycotoxin doses may remain undetected.

Flow cytometry is an indispensable analytical method in contemporary immunological research. This technique supports the detection of subtle changes in the size of various subpopulations of immunocompetent blood cells. Flow cytometry is also used to determine the number of cells characterized by complex immunophenotypes, such as dendritic cells (DC). A study of DON’s (12 µg/kg BW) effects on CD4^+^8^−^, CD4^+^8^+^, and CD4^−^8^+^ peripheral blood lymphocytes revealed that this mycotoxin disrupts the growth of CD4^+^8^+^ cells [23]. The above experiment was performed on animals of different genotypes, with the use of synthetic DON. The subtle influence of DON on the immune system was best reflected in a disrupted linear increase in the percentage of double positive (DP) cells. In contrast, Ferrari et al. [24] reported that a DON dose of 0.5 ppm per pig did not induce any changes in the subpopulations of CD3^−^CD8^+^, CD4^+^CD8^−^, CD4^−^CD8^+^, CD8^high^, and CD4^+^CD8^+^ lymphocytes. However, the cited studies involved farm animals characterized by high individual variation, and chemically-synthesized DON was used. In a study by Döll et al. [25], pigs receiving DON (5.7 mg/kg of feed) did not exhibit changes in the subpopulations of CD3^+^, CD4^+^, and CD8^+^ cells or IgA^+^ splenic cells relative to the control group. Additionally, in this case, the authors pointed to the fact that the effects exerted by DON may vary depending on the time of exposure, and a four-week experiment could be too short to observe changes in the immune system. Different results were noted in a study of broiler chickens whose diets were contaminated with DON and ZEN (8.2 and 8.3 mg/kg of feed, respectively). The administered mycotoxin doses lowered the percentage of CD3^+^ cells, i.e., the entire population of T cells [26]. In a study of rats, exposure to nivalenol (NIV), a type B trichothecene, modified the proportions of CD4^+^ and CD8^+^ lymphocytes and increased the CD4^+^/CD8^+^ ratio [27].

Dendritic cells are a very important subgroup of immunocompetent cells responsible for antigen presentation and modulation of the immune response [28]. Two types of DCs have been identified: type 1 interferon-producing cells (plasmacytoid dendritic cells, pCDs) and conventional dendritic cells (cCDs). Both DC types are found in lymphoid and non-lymphoid tissues and in blood [29]. To date, DON’s effect on porcine DCs has not been studied in vivo. Earlier experiments evaluating DON’s effects on DCs were performed in vitro. Dendritic cells were obtained by stimulating monocytes or marrow cells with the use of the granulocyte-macrophage colony-stimulating factor (GM-CSF) and IL-4. In previous research, the observed decrease in the viability of DCs was determined by the dose of the mycotoxin and the period of exposure. Deoxynivalenol also disrupts the maturation of DCs by compromising the expression of markers such as CD86, HLA-DR, and CCR7 [30]. In an in vitro experiment performed on DCs harvested from BALB/c mice, DON inhibited cell maturation by blocking the expression of major histocompatibility complex class II (MHC II) and CD11c. The ability of DCs to secrete IL-12 and IL-10 was also compromised under exposure to DON [31]. In an in vitro study of porcine DCs induced from monocytes, DON disrupted the expression of CD80/86 and CD40 and activated MAP, ERK1/2, and JNK kinases [32].

Research on DON’s influence on the function of the immune system often focuses on changes in the secretion of proinflammatory and anti-inflammatory cytokines. The results are used to identify the type of immune response to mycotoxin exposure. The available data suggest that DON stimulates IL-4 secretion. Meky et al. [33] demonstrated that human lymphocytes incubated with DON and stimulated with phytohemagglutinin (PHA) produced more IL-4. According to Lessard et al. [34], animals receiving feed contaminated with DON have elevated IL-4 mRNA levels in the jejunum. IL-4 induces the polarization of naive cells to the Th2 phenotype, which activates the humoral immune response [35]. These results suggest that feed contaminated with DON led to the polarization of the humoral immune response, as confirmed by a decrease in the population of CD4-8+ cells, i.e., cytotoxic lymphocytes that recognize antigens presented by MCH class I molecules. The population of CD4+8- cells increased, which could result from the differentiation of naive cells to the Th2 phenotype. IL-4 is also involved in the induction of dendritic cells. In the work of Basak et al. [36], continuous infusion of rIL-4 and rGM-CSF at 10 µg/day increased the counts of peripheral DC in C57BL/6 mice. The above experiment demonstrated that DC are induced by the combined effect of both cytokines. Choi et al. [37] administered an aqueous solution of DON (2 mg/L) to C3H mice and observed an increase in the thymic concentrations of GM-CSF and IL, as well as an increase in cDC and pDC counts. The increase in pDC counts is particularly interesting because this cell population induces a Th2 response [38]. The above results suggest that prolonged exposure to DON can polarize the immune system towards a humoral response in closely-related animals.

## 4. Materials and Methods

All experimental procedures involving sample collection and storage were carried out in compliance with decision No. 45/2013 of 19 September 2013 of the Local Ethics Committee in Olsztyn.

### 4.1. Animals

The experiment was performed on eight crossbred (Polish Large White × Polish Landrace) weaners from the same litter, with an average initial body weight of 24.3 ± 1.09 kg. After one week of adaptation, the animals were divided into two equal groups: a control group (C, *n* = 4) and an experimental group (M, *n* = 4). Both groups consisted of two males and two females. The groups were kept in an experimental pig house, in two separate pens (4 m × 4 m) equipped with automatic drinkers to ensure free access to water. The pig house had a mechanical ventilation system with HEPA filters. The following microclimatic conditions were maintained throughout the experiment: air temperature—21 °C, relative humidity—65%, air flow rate—0.2 m/s. Feed contaminated with DON was supplied by a commercial feed mill whose representatives performed a mycotoxicological analysis in the laboratory of the Department of Veterinary Prevention and Feed Hygiene, Faculty of Veterinary Medicine, University of Warmia and Mazury in Olsztyn, as part of owner supervision. The feed administered to the control group animals was supplied by the same producer, and it was subjected to mycotoxicological analysis before the experiment. Feed was provided twice daily, at 8:00 a.m. and 5:00 p.m., in loose form, at a dose of 850 g (2 × 850 g = 1700 g per day) throughout the experiment. The composition of the complete diet, declared by the manufacturer, is presented in Table 4. At the end of the six-week experiment, blood was sampled from every animal for laboratory analyses.

### 4.2. Body Weights of Pigs

The animals from both groups were weighed at the beginning (week 0) and at the end (week 6) of the experiment, with the use of a Radwag WPT/4 300 C6 platform weighing scale (Radwag, Radom, Poland).

### 4.3. Mycotoxin Levels in Feed

DON was extracted from experimental feed on immunoaffinity columns (DON-Test^TM^ Don Testing System VICAM, Watertown, MA, USA). All extraction procedures were performed in accordance with the recommendations of the column manufacturer. Chromatographic analyses were conducted with the use of an Agilent (Santa Clara, CA, USA) 1100 series HPLC system and 4.6 × 100 mm (3.5 μm) Eclipse Plus C^18^ columns. The mobile phase was a water and acetonitrile mixture with a 90:10 solvent ratio. The flow rate was 0.6 mL/min. The mycotoxin was identified with the use of a diode detector at a wavelength of 220 nm.

The presence of zearalenone (ZEN), fumonisins B_1_ and B_2_ (FB_1_ and FB_2_), and ochratoxin A (OTA) was determined by methods described in the literature [39,40,41].

### 4.4. Proximate Chemical Composition of Feed

The proximate chemical composition of diets fed to pigs in groups C and M were determined using the NIRS™ DS2500 F feed analyzer (FOSS, Hillerød, Denmark), which is a monochromator-based NIR reflectance and transflectance analyzer with a scanning range of 850–2500 nm.

### 4.5. Hematology Tests

Blood samples of 2 mL were collected from pre-pubertal pigs into test tubes containing EDTAK2 (Sigma Aldrich, Saint Louis, MO, USA) as an anticoagulant. The samples were thoroughly mixed and analyzed to determine RBC, MCV, MCHC, mean corpuscular hemoglobin (MCH), concentration of hemoglobin in whole blood (HGB), HCT, PLT, red cell distribution width (RDW), hemoglobin distribution width (HDW), MPV, WBC, including neutrophils (NEUT), lymphocytes (LYMPH), monocytes (MONO), eosinophils (EOS), basophils (BASO), and immature leukocytes (LUC). The above parameters were determined in a Medonic hematology analyzer according to the procedure recommended by the manufacturer. Whole blood in EDTAK2 tubes was analyzed by flow cytometry and laser scanning in a Siemens Advia 2120i hematology analyzer equipped with (i) an optical peroxide biosensor for measuring dispersed light and light absorbed by individual cells by hydrodynamic focusing on a cell stream in a flow-through cuvette; (ii) laser optics for measuring high-angular and low-angular light dispersion and absorption by individual cells, where the laser diode was the source of light (the measurement was performed to evaluate red blood cells, platelets, and lobulation of nuclei in white blood cells); (iii) a hemoglobin colorimeter, for measuring the lamp voltage corresponding to the amount of transmitted light; and (iv) PEROX and BASO reagents, for generating differential cytograms.

### 4.6. Cytometric Analysis

Blood samples for cytometric analyses were collected into test tubes containing EDTAK2. The samples were thoroughly mixed with the anticoagulant before analysis.

#### Determination of the Percentages of Lymphocytes (CD4^+^8^−^, CD4^−^8^+^, CD4^+^8^+^), Monocytes, and DCs

To determine the percentages of lymphocytes, 50 µL blood samples were transferred to cytometry tubes. They were combined with mouse anti-pig monoclonal antibodies specific for CD4 (CD4a:FITC, clone MIL17) (AbD Serotec, UK) and CD8 (CD8a:PE, Clone 76-2-11) (BD, Franklin Lakes, NJ, USA) receptors on T cells, in quantities recommended by the manufacturer. Blood samples were incubated on ice for 30 min. After incubation, red blood cells were lysed with the FACS^TM^ lysing solution (BD, Franklin Lakes, NJ, USA) for 12 min, at room temperature, in darkness. The remaining cells were rinsed with PBS and centrifuged at 300× *g* for 5 min at room temperature. The analysis of cDC (CD172a^high^4^−^14^−^), pDC (CD172a^dim^4^+^14^−^) and monocyte (CD14^+^) phenotypes was performed according to the gating strategy presented by Ravlo et al. [42]. Blood samples (100 µL) were transferred to cytometry tubes. They were combined with monoclonal antibodies against surface antigens characteristic of porcine dendritic cells and peripheral blood monocytes: mouse anti-pig (AbD Serotec, Oxford, UK) specific for antigens CD172a (CD172a: RPE, clone BL1H7) and CD14 (CD14: FITC, clone MIL2) and mouse anti-pig (BD, Franklin Lakes, NJ, USA) CD4a: PerCP-Cy^TM^5.5 (clone 74-12-4). Antibodies were added in quantities recommended by the manufacturer, and they were incubated on ice for 30 min. After incubation, red blood cells were lysed with the FACS lysing solution (BD, Franklin Lakes, NJ, USA) for 12 min, at room temperature, in darkness. The remaining cells were rinsed with PBS and centrifuged at 300× *g* for 5 min at 5 °C.

**Figure 3:**
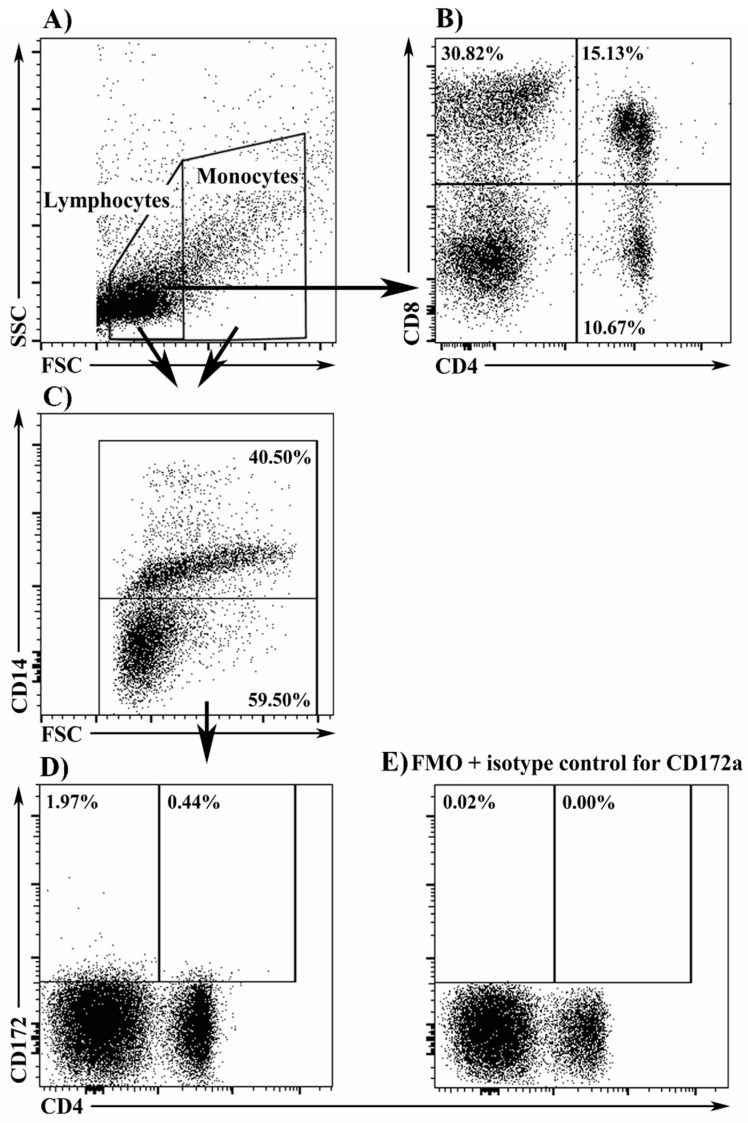
(**A**) The entire PBMC population was gated and a lymphocyte gate was set in the FSC vs. SSC plot; (**B**) Lymphocytes expressing the analyzed antigens were isolated in the CD4 vs. CD8 plot; (**C**) CD14^+^ (monocytes) and CD14^−^ cells were isolated from PBMC in the FSC vs. CD14 plot; (**D**) cDC (CD172a^high^4^−^) and pDC (CD172a^dim^4^+^) were isolated in the CD4 vs. CD172a plot; (**E**) The Fluorescence Minus One (FMO) plus isotype control for CD172a antibody was used to determine the gate for the CD172a^+^ cells.

The samples were analyzed with the use of the FACSCanto II flow cytometer (BD, Franklin Lakes, NJ, USA). Data on the percentages of lymphocytes (CD4^+^8^−^, CD4^−^8^+^, CD4^+^8^+^), monocytes, and DCs were acquired by recording 30,000 events for each sample. The data were analyzed using FlowJo software (Version 8.8.6, Tree Star, Inc., Ashland, OR, USA). Lymphocytes were identified based on forward- and side-scatter (FSC/SSC) properties and then gated for expression of CD4 and CD8 surface receptors. The peripheral blood mononuclear cell (PBMC) fraction was isolated in the FSC vs. CD14 plot to identify DC. The populations of CD14^−^ (DC) and CD14^+^ (monocyte) cells were isolated in the FSC vs. CD14 plot. CD14^−^ cells were analyzed for CD4 and CD172a expression. CD172a^high^4^−^14^−^ cells were classified as cDC, and CD172a^dim^4^+^14^−^ cells as pDC. CD172a cells were stained with Fluorescence Minus One controls because CD172a^−^ cells were smoothly transformed to the CD172a^+^ lineage, which obstructed CD172a^+^ gating. The Fluorescence Minus One control for CD172a was performed according to the same protocol, but CD172a (CD172a:RPE, clone BL1H7) was replaced with mouse IgG1 isotype control (clone MCA928) (AbD Serotec, Bio-Rad, Oxford, UK) (Figure 3).

### 4.7. Statistical Analysis

Data were processed statistically in the Statistica program (StatSoft Inc., Tulsa, OK, USA). The results were analyzed by the Shapiro-Wilk test to test the assumption of normal distribution in groups M and C. Differences between groups were determined by the Student’s *t*-test. The results were regarded as statistically significant at *p* ≤ 0.05.

## 5. Conclusions

The results of this study suggest that feed naturally contaminated with low doses of DON has immunomodulatory properties, polarizing the immune response towards humoral immunity. Further research into the effects exerted by low doses of DON should involve inbred animals and focus on changes in the proportions of immunocompetent cells in view of the altered cytokine profile.

## Figures and Tables

**Table 1:** Proximate chemical composition of pig diets.

	Groups	C	M
Parameter			
Ash (g/kg)		50.60	51.20
Crude fiber (g/kg)		45.95	43.09
Starch (g/kg)		456.90	441.50
Protein (g/kg)		162.95	167.10
Fat (g/kg)		41.90	43.00
Moisture content (%)		11.97	12.01

**Table 2:** White blood cell counts, platelet counts, and mean platelet volume in the peripheral blood of pigs from groups C and M.

	Parameter	WBC 10^3^/μL	NEUT 10^3^/μL	LYMPH 10^3^/μL	MONO 10^3^/μL	EOS 10^3^/μL	BASO 10^3^/μL	LUC 10^3^/μL	PLT 10^3^/μL	MPV fL
Groups										
C	Mean	14.40	3.97	9.43	0.60	0.09	0.10	0.21	310.25	9.08
	SD	3.52	0.51	3.40	0.14	0.05	0.04	0.14	65.06	1.51
M	Mean	11.65	3.04	7.82	0.42	0.09	0.07	0.22	346.75	9.00
	SD	1.68	0.98	0.86	0.05	0.09	0.04	0.16	96.73	1.32

Abbreviations: white blood cells (WBC), neutrophils (NEUT), lymphocytes (LYMPH), monocytes (MONO), eosinophils (EOS), basophils (BASO), leukocytes (LUC), platelet (PLT), mean platelet volume (MPV).

**Table 3:** Erythrogram of the peripheral blood of pigs from groups C and M.

	Parameter	RBC 10^6^/μL	HGB g/dL	HCT %	MCV fL	MCH pg	MCHC g/dL	RDW %	HDW g/dL
Groups									
C	Mean	6.88	11.50	36.58	53.15	16.78	31.53	16.80	1.60
	SD	1.01	1.62	6	1.83	1.16	1.65	1.47	0.15
M	Mean	6.89	11.33	36.80	53.40	16.48	30.88	15.40	1.57
	SD	0.97	1.43	5.32	0.68	0.64	1.04	0.92	0.10

Abbreviations: red blood cells (RBC), concentration of hemoglobin in whole blood (HGB), hematocrit (HCT), mean corpuscular volume (MCV), mean corpuscular hemoglobin (MCH), mean corpuscular hemoglobin concentration (MCHC), red cell distribution width (RDW), hemoglobin distribution width (HDW).

**Table 4:** Declared composition of the complete diet.

INGREDIENTS	Composition of the Complete Diet, Declared by the Manufacturer (%)
Soybean meal	16
Wheat	35
Barley	42.3
Wheat bran	4.0
Limestone	0.2
Mineral-vitamin premix ^1^	2.5

^1^ Composition per kg: vitamin A: 500,000 IU, iron: 5000 mg, vitamin D_3_: 100,000 IU, zinc: 5000 mg, vitamin E (alpha-tocopherol): 2000 mg, manganese: 3000 mg, vitamin K: 150 mg, copper (CuSO_4_·5H_2_O): 500 mg, vitamin B_1_: 100 mg, cobalt: 20 mg, vitamin B_2_: 300 mg, iodine: 40 mg, vitamin B_6_: 150 mg, selenium: 15 mg, vitamin B_12_: 1500 μg, l-lysine: 9.4 g, niacin: 1200 mg, dl-methionine+cystine: 3.7 g, pantothenic acid: 600 mg, l-threonine: 2.3 g, folic acid: 50 mg, tryptophan: 1.1 g, biotin: 7500 μg, phytase+choline: 10 g, ToyoCerin probiotic+calcium: 250 g, antioxidant+mineral phosphorus and released phosphorus: 60 g, magnesium: 5 g, sodium: 51 g.
